# Supplementary material for: Benchmarking substrate-based kinase activity inference using phosphoproteomic data
Source: Bioinformatics. 2017 Feb 13;33(12):1845–51. doi: 10.1093/bioinformatics/btx082 (PMC5870625; doi:10.1093/bioinformatics/btx082)
Supplement: Supplementary Data [file btx082_supp.zip › Supplementary Figures.pdf]

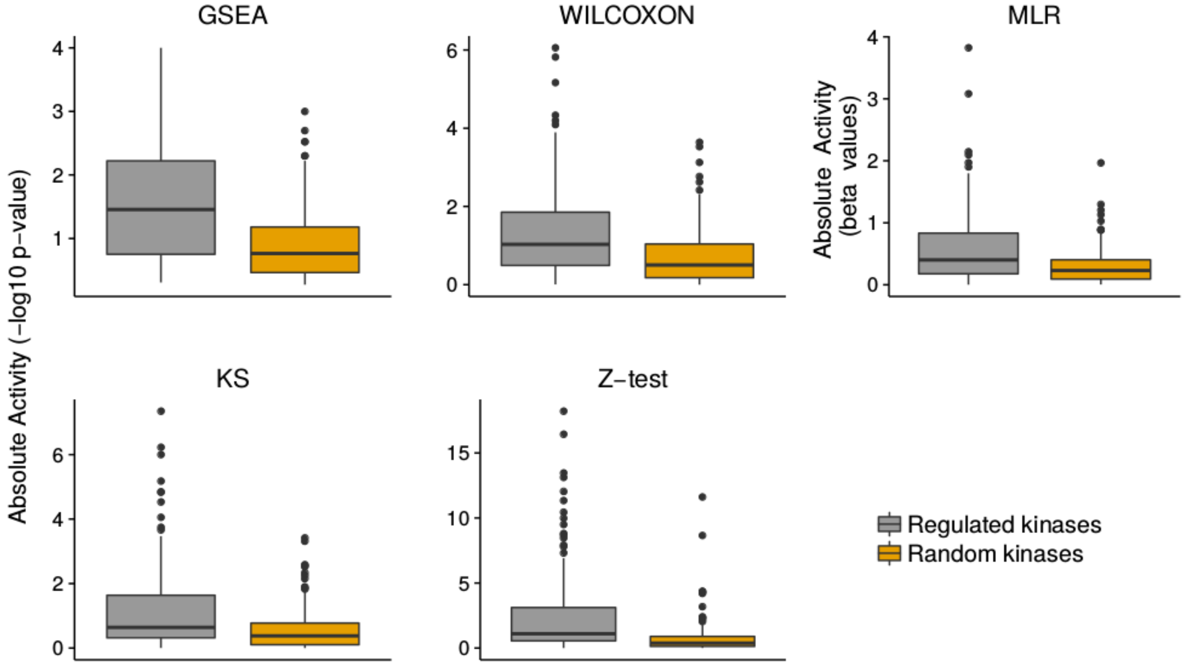

Supplementary Figure S1. Predicting kinase regulation using 5 substrate-based methodologies. Absolute activity scores for 149 kinase-conditions expected to show regulation are compared against the same number of randomly generated pairs using the 5 methodologies under comparison.

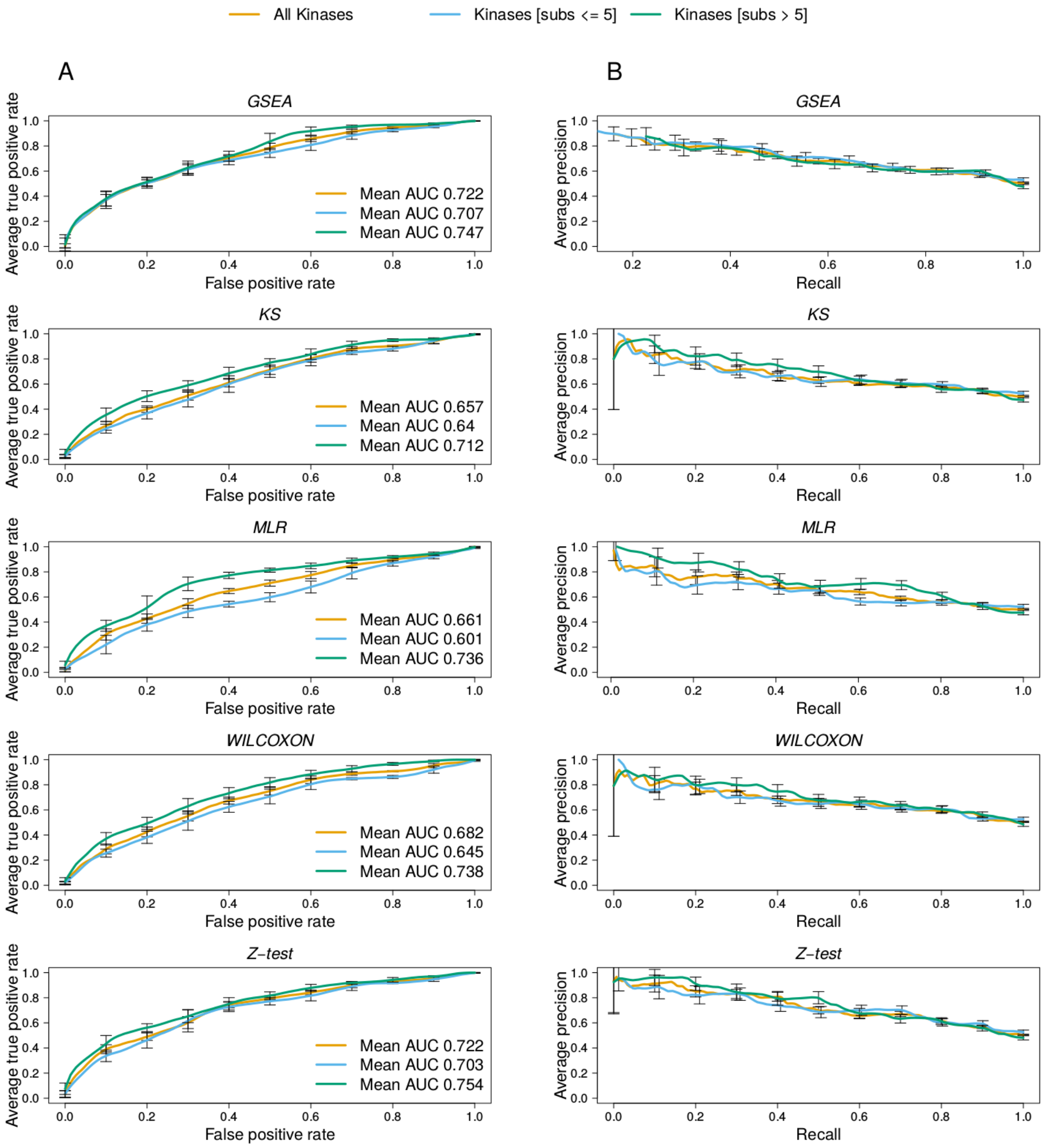

Supplementary Figure S2. ROC and Precision-Recall (PR) curves comparing kinase activity prediction methods performance per number of known substrates. The kinase activity scores were calculated using the -log10 of the p-values of each statistic, with the exception of MRL, and were signed based on the mean phosphorylation value of the substrates set. For the MRL model the beta values were used as the estimation of the regulation of each kinase. A total of 149 kinase-conditions cases are present in the positive set and were paired with 60 negative sets of 149 independently sampled random kinase-conditions. (A) The ROC and (B) PR curves were calculated for the whole benchmark and by grouping the kinases in two categories: few (<= 5 substrates) and many substrates (> 5 substrates). The average curves of the 60 validation sets are shown for each comparison.

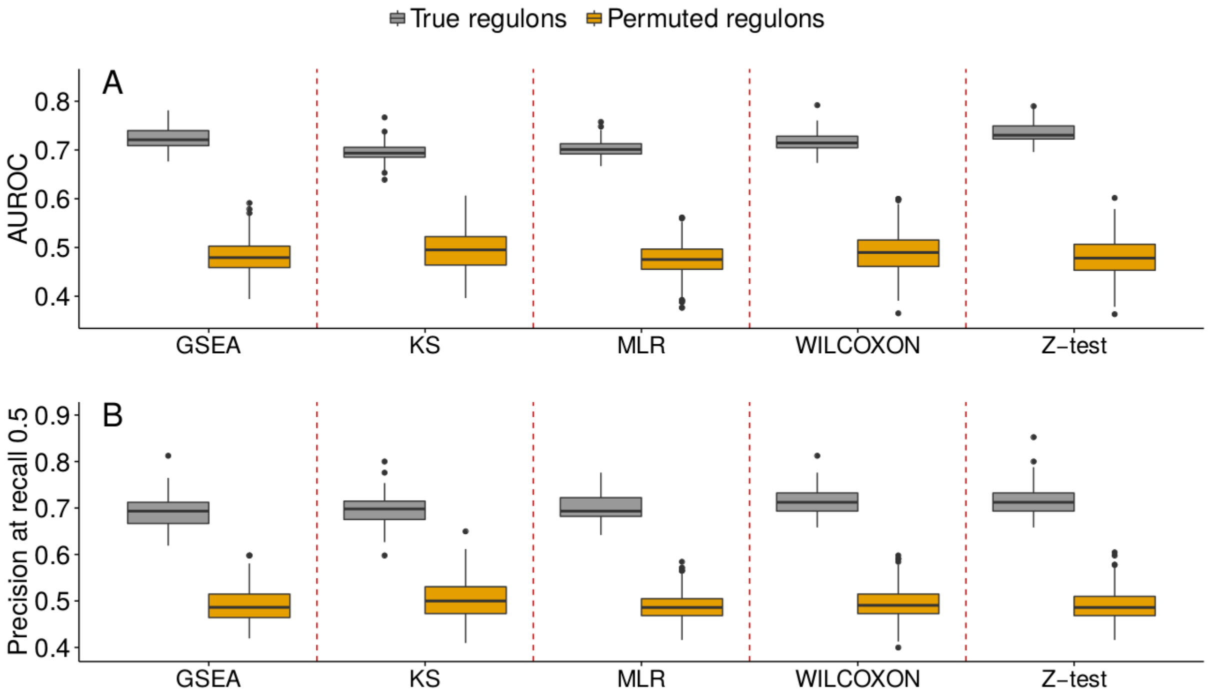

Supplementary Figure S3. Methods performance comparison using the true and permuted sets of kinase-substrates interactions. A total of 10 permuted sets of kinase-substrate relationships conserving their original degrees of connectivity were generated to test the accuracy of the methods to random groups of substrates. The performance metrics of the methods were compared against the true kinase-substrates interactions. Only 105 positive cases of the benchmark described in Table 1 were considered due to missing quantifications of substrates in the permuted sets. This set was compared against 60 randomly generated negative sets to calculate the ROC and Precision-Recall curves. AUC values of the ROC curves (up) and precision values at recall 0.5 (down).

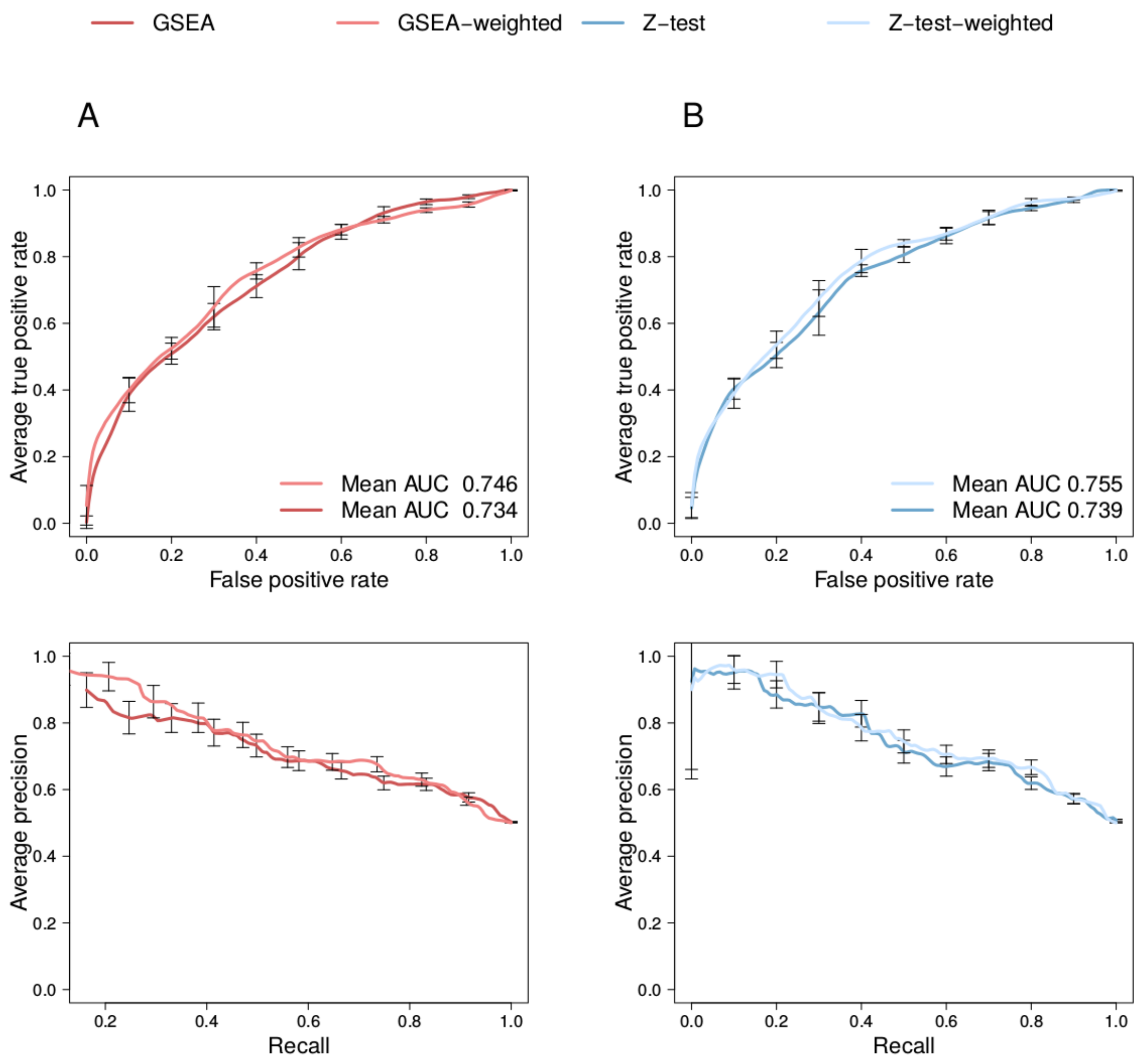

Supplementary Figure S4. ROC and Precision-Recall (PR) curves for the weighted vs unweighted GSEA and Z-test. Same data as Figure 2. ROC – top – and PR – bottom – curves for GSEA (A) and Z-test (B). The average curves of the 60 validation sets are shown for each method. A total of 135 kinase-conditions cases are present in the positive set and were paired with 60 random negative sets sampled independently.

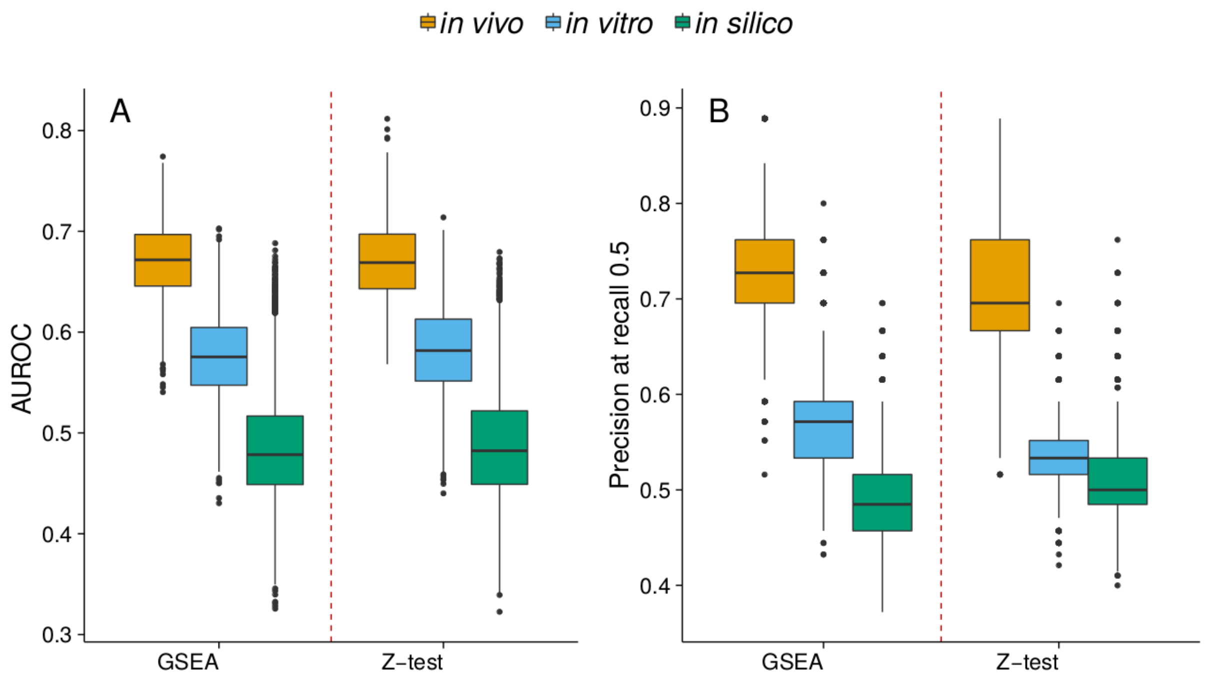

Supplementary Figure S5. As Figure 3 but downsampling the substrates. Benchmark of kinase activity predictions using *in vivo*, *in vitro* or *in silico* supported substrates. (A) AUC performance. (B) Precision at recall 0.5. Positive kinase-condition pairs are 31 in all sets. Kinase-substrates collections were down sampled 25 times to the size of the smallest set.

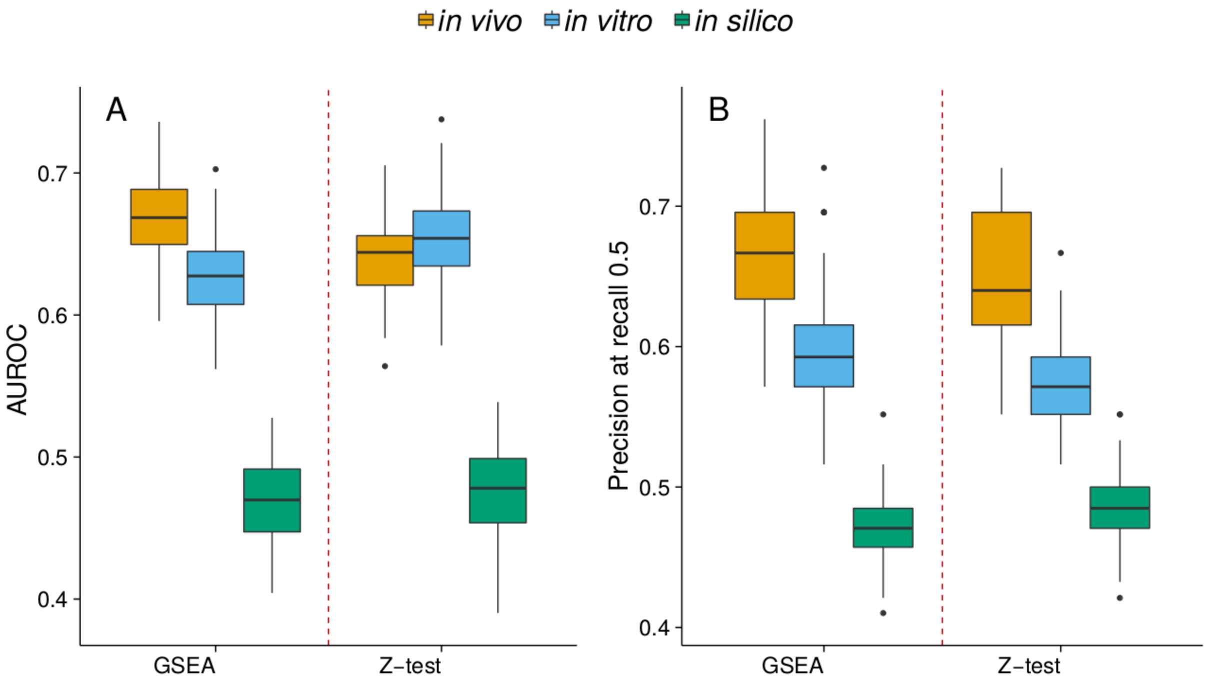

Supplementary Figure S6. As Figure 3 but having the same positives in all sets. Benchmark of kinase activity predictions using *in vivo*, *in vitro* or *in silico* supported substrates. (A) AUC performance. (B) Precision at recall 0.5. Positive kinase-condition pairs are 31 in all sets.

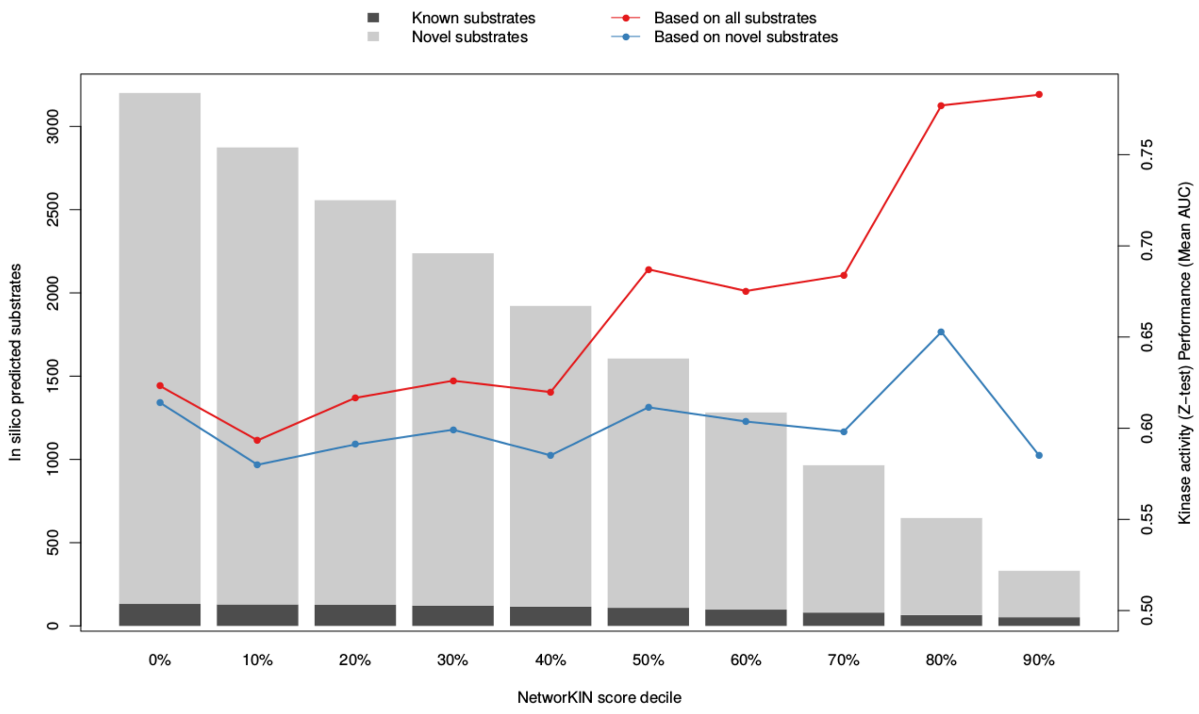

Supplementary Figure S7. Effect of NetworKIN score in the performance on kinase activity predictions based on *in silico* supported substrates. NetworKIN predicted substrates are discretized per kinase using score deciles. Total number of known and previously reported NetworKIN predicted substrates are visualized in the barplot - left axis. Performance of the predictions measured as mean AUC of the Z-test predictions is displayed for all predicted substrates - red - and for all predicted substrates not previously reported - blue.

Supplementary Tables

Table S1. Description of studies and biological perturbations included in gold standard.
